# Pathway-based analyses

**DOI:** 10.1186/s12863-015-0314-9

**Published:** 2016-02-03

**Authors:** Jack W. Kent

**Affiliations:** Department of Genetics, Texas Biomedical Research Institute, PO Box 760549, San Antonio, TX 78245-0549 USA

## Abstract

**Background:**

New technologies for acquisition of genomic data, while offering unprecedented opportunities for genetic discovery, also impose severe burdens of interpretation andpenalties for multiple testing.

**Methods:**

The Pathway-based Analyses Group of the Genetic Analysis Workshop 19 (GAW19) sought reduction of multiple-testing burden through various approaches to aggregation of highdimensional data in pathways informed by prior biological knowledge.

**Results:**

Experimental methods testedincluded the use of "synthetic pathways" (random sets of genes) to estimate power and false-positive error rate of methods applied to simulated data; data reduction via independent components analysis, single-nucleotide polymorphism (SNP)-SNP interaction, and use of gene sets to estimate genetic similarity; and general assessment of the efficacy of prior biological knowledge to reduce the dimensionality of complex genomic data.

**Conclusions:**

The work of this group explored several promising approaches to managing high-dimensional data, with the caveat that these methods are necessarily constrained by the quality of external bioinformatic annotation.

## Background

The data provided for Genetic Analysis Workshop 19 (GAW19) offer extensive and complex genomic information (described fully in Blangero et al. [1]). These data included 2 Mexican American cohorts: a set of extended families providing longitudinal data on blood pressure (BP; up to 4 clinic visits per subject) and genomic data including haplotype-tagging single nucleotide polymorphisms (SNPs) and whole sequence data for odd-numbered autosomes, as well as gene expression profiles from the first clinic visit; and a separate set of unrelated individuals with BP and exome sequence data from a single clinic visit. The GAW19 organizers also provided 200 replicates of simulated BP data for both cohorts based on the real genotypes and a polygenic generating model using functional variants in genes chosen based on real associations between BP and gene expression phenotypes in the Type 2 Diabetes Genetic Exploration by Next-generation sequencing in Ethnic Samples (T2D-GENES) study ([1]). This rich genomic resource serves as a sampler of the types of data now becoming available for many study cohorts as a result of rapid technological advances (and attendant decreased costs) in acquisition of genomic information.

For GAW19, as in all such studies, this abundance is both a blessing and a curse, a potential source of new insights into the mechanisms of complex disease phenotypes that also introduces an unprecedented burden of correction for multiple tests and interpretation of results. A class of emerging techniques for managing this abundance involves narrowing the search space by grouping the units of analysis (gene expression probes, sequence variants, etc) into biologically relevant pathways (see, eg, Wang et al [2] for a review of software for pathway analysis to follow genome-wide association studies [GWAS]). Pathway analysis can proceed along at least 2 general lines [3]:

1. Preselection of biological pathways believed to be relevant to the disease or phenotype of interest. The primary goals of this approach are to limit the number of genomic features, and therefore the multiple-testing burden, to those annotated to genes in the candidate pathways, and/or to confirm the prior biological hypotheses. An additional aim is to manage genetic heterogeneity: for example, different lineages may segregate variation in different genes in the same pathway that nonetheless yield similar biological effects. Without prior biological knowledge, this heterogeneity might be interpreted as noise rather than as concordant signal [4].
2. Gene enrichment tests of genomic features prioritized by evidence of association with the phenotype of interest to identify biological pathways a posteriori. The primary goal of this approach is to interpret the biological significance of findings from agnostic tests of association.

Both of these lines of investigation are constrained by existing biological knowledge (and by the curation strategies and quality of available bioinformatic databases).

Because pathway analysis is relatively new, methods for assigning genomic features to pathways and for testing the significance of these assignments in relation to phenotypes are still very much under development. This developmental fluidity was the context for this discussion group at GAW19.

## Methods

This report is based on the work of 7 research teams who presented their work in our group discussion at GAW19. In the following discussion, these teams are referenced by the name of the presenting author, as shown in Table 1.

**Table 1:** Research teams participating in the pathway-based analyses group

Presenting author	Abstract Title/Coauthors	Reference
Brunel H	Meta-expression: A methodology for the joint analysis of gene expression in biological pathways. Massanet R, Fernández-Albert F, Ziyatdinov A, Soria JM, Perera A	Unpublished
Kos MZ	Comparison of GWAS and exome sequence data sets from GAW19. Blackburn AN, Almasy L	Unpublished
Lo A	Network-guided interaction mining for blood pressure phenotype of unrelated individuals in GAW19. Agne M, Auerbach J, Fan R, Lo S-H, Wang P, Zhang T	Unpublished
Quillen EE	A variance component method for integrated pathway analysis of gene expression data. Blangero J, Almasy L	[13]
Tayo BO	Association of polymorphisms in the aldosterone-regulated sodium reabsorption pathway with blood pressure among Hispanics. Tong L, Cooper RS	[11]
Valcarcel A	A hierarchical approach to SNP-set analysis: An evaluation of power and type 1 error of gene-based tests of association after pathway-based analysis. Grinde K, Cook K, Green A, Tintle N	[9]
Ziyatdinov A	Prioritization strategies in enrichment analysis of gene expression data in GAW19 family-based study. Fernández-Albert F, Vazquez-Santiago M, Soria JM, Perera-Lluna A	unpublished

Tables 2 and 3 summarizes the teams’ approaches to the group topic, including the focal phenotypes chosen from the GAW19 data, the focal genomic feature (gene expression or genetic variants), and the analytical tools employed. These choices, which were intimately associated with each team’s primary aims, are compared and discussed in detail under Results.

**Table 2:** Summary of research approaches: Data used

Contribution	Genomic data	Cohort	Phenotype category	Phenotype(s)
Brunel	Gene expression/GWAS	Families	Real (gene expression)	Derived^a^
Kos	GWAS and exomic variants	Families	Simulated^b^	DBP (first visit)
Lo	GWAS and exomic variants	Unrelated	Real	DBP (first visit)
Quillen	Gene expression	Families	Simulated^b^	DBP (first visit)
Tayo	GWAS and exomic variants	Unrelated	Simulated^b^ and real	DBP, SBP, HT, MAP
Valcarcel	GWAS and exomic variants	Families	Simulated^b^	Means across visits of DBP, SBP, HT, MAP
Ziyatdinov	Gene expression	Families	Real	SBP

*DBP* diastolic blood pressure; *GWAS*, genome-wide association studies; *HT* hypertension; *MAP* mean arterial pressure; SBP, systolic blood pressure

^a^Independent components (see Table 3 and text)

^b^Authors were aware of simulation model “answers”

**Table 3:** Summary of research approaches: Analytical strategies

Contribution	Pathway assembly	Probe/variant filters	Source of annotations/ biological significance	Analytical tools
Brunel	Annotated to BP genes and interactors	Ontology, protein-protein interaction	Gene Ontology, other (unspecified)	Independent components analysis, GWAS in GenABEL
Kos	Synthetic	Nonsynonymous or stop codon gain/loss	KEGG, Biocarta, Pathway Interaction Database, Reactome	SOLAR measured genotype association
Lo	Annotated to type 2 diabetes genes and interactors	Not applicable	OMIM, GeneMania	Bespoke method to detect SNP-SNP interaction
Quillen	Synthetic	Not applicable	Ariadne Pathway Studio 8.0	Empirical similarity matrices, variance decomposition
Tayo	Annotated to genes in specific pathway	BP-related pathway of interest	KEGG	PLINK association
Valcarcel	Synthetic	Not reported	ANNOVAR	SKAT, burden tests
Ziyatdinov	Associated with BP	Heritability	Gene Ontology	Linear mixed models

*ANNOVAR* Annotate Variation; *BP* blood pressure; *GWAS* genome-wide association studies; *KEGG* Kyoto Encyclopedia of Genes and Genomes; *OMIM* Online Mendelian Inheritance in Man; *SKAT* sequence kernel association test; *SNP* single nucleotide polymorphism; *SOLAR* Sequential Oligogenic Linkage Analysis Routines

## Results and discussion

### Motivation

An overriding motivation for employing pathway analysis was to reduce the multiple-testing burden by basing inferences on the combined effects of probes or variants. Most teams used external bioinformatic data to inform pathway construction (see Table 3). However, the use of pathways to clarify biological function or interpret association results, while highly relevant to future applications of these methods, was a secondary concern given the method-development focus of these studies. Of all the studies, Ziyatdinov came closest to this interpretive approach, using relationships in the data to recover the biological processes implicit in the simulation generating model.

### Data selection; pathway assembly

All teams used the BP data (real or simulated) provided for GAW19, including systolic (SBP) and diastolic BP (DBP) and hypertension (HT) status. Several teams also calculated the derived phenotype mean arterial pressure (MAP) from these data as (2/3 DBP + 1/3 SBP). All teams using the simulated data chose not to be blind to the generating model (they “used the answers”), which allowed them to estimate power to detect “true” functional variants and associated gene expression phenotypes. Most teams also used the provided null trait Q1 to estimate type 1 error rate. A limitation of this use of the simulated data (as noted by a reviewer of an early draft of this report) is that evaluation of method performance is necessarily sensitive to the assumptions of the generating model.

Three teams (Brunel, Quillen, Ziyatdinov) focused on the gene expression probes, while the remaining teams considered single nucleotide variants (SNVs) drawn from the GWAS genotypes, exome sequence data, or both. Combined analysis of gene expression and association has been considered in previous GAWs (eg, Charlesworth et al. [5]), but only Brunel attempted this combination in a 2-step approach (data reduction of BP-related gene expression phenotypes followed by GWAS).

As noted, most teams made use of the simulated phenotypes as a basis for estimating power and type 1 error. Kos, Quillen, and Valcarcel constructed synthetic pathways (described more fully below) of gene expression probes (Quillen) or SNVs (Kos, Valcarcel) representing predetermined numbers of randomly chosen genes; these synthetic pathways were then characterized by the number of genes that contributed to the simulated phenotypes as a basis for measuring the effect of functional “dosage” on analytical power. One team (Tayo) focused on a single biological pathway with known relevance to hypertension (aldosterone-regulated sodium reabsorption), using both real and simulated BP phenotypes.

Kos, Quillen, and Valcarcel independently conceived of using sets of probes or variants annotated to randomly selected genes to define *synthetic pathways* (SPs) to test both the power and type 1 error of their proposed methods. SPs that represented genes used in the simulation generating model were, in Quillen’s terminology, “positive controls” to test the sensitivity of tests. SPs lacking such representation provided data on false positive rate, either in place of or supplementary to tests of the null trait Q1. All 3 teams used various metrics—for example, number of genes represented in each SP or number of generating-model genes represented—to further characterize the performance of their analytical approaches.

In addition to the dimensional reduction achieved by grouping probes or variants in pathways, some teams also filtered variants based on predicted function (eg, Kos).

Lo developed biological pathways nominally based on a hypothesis that hypertension could be related to undetected type 2 diabetes (this is known to have elevated prevalence in the Mexican American cohorts from which the GAW19 data were derived, but diabetes status was not reported as part of the provided data set). Pathway construction consisted of choosing a set of diabetes-related genes from the literature and then expanding these “seed” genes with publicly available gene interaction data. In the context of this workshop, this approach simply provided a biologically plausible way to construct pathways reflecting the number of genes and patterns of gene–gene interaction that might be encountered in a “real” study; this was tangential to the primary goal of characterizing performance of these investigators’ bespoke method for analyzing SNP-SNP interaction effects [6] (see below).

As noted, most pathways were user-defined, either based on numbers of genes (to test effects on power and specificity) or on prior biological information, rather than on empirical networks (eg, by weighted correlation networks) [7]. This was consistent with the emphasis on methodological development, especially on data reduction and control of the burden of multiple testing. The known causal structure of the simulated data might have supported a test of the efficacy of an empirical networks approach to recover the generating model from patterns of SNV association, but no team attempted this.

### Analytical tools

The focus for methodological innovation in these studies was typically on the construction or definition of pathways. In general, established tools were used for relating pathway-defined probes or variants to the traits of interest, accounting for family data where appropriate: for example, variance components-based analyses implemented in SOLAR (Sequential Oligogenic Linkage Analysis Routines) [8], by Kos and Quillen; adaptation of SKAT (sequence kernel association test) by Valcarcel; burden tests [9]; GenABEL [10] by Brunel; and PLINK for unrelated data [11].

An exception to this pattern was the use by Lo of an analytical tool to detect gene interactions, previously developed by members of this team [6]. In brief, this method involves comparing the joint effect of 2 SNPs on a trait to the marginal effect of each; a ratio of joint to marginal effects significantly greater than 1 (as assessed by permutation) is taken as evidence of SNP–SNP interaction effects on the phenotype [6]. Using pairwise tests of SNPs annotated to genes in a pathway of interest (described above), these authors reported evidence of a joint effect of variants in *GCK* and *PAX4* on hypertension (real data from the unrelated cohort). As noted by the anonymous reviewers of these authors’ report, the nature of this joint effect—whether due to in-sample LD, interaction between the gene products, or some other effect—needs additional investigation.

### Gene expression

We now consider selected approaches in more detail. Taking replicate simulations of DBP as the trait of interest, Quillen analyzed the random effect of similarity in gene expression among related individuals, with the goal of identifying pathways for which coexpression accounted for a significant proportion of variance relative to modeling the random effect of kinship alone. This analysis compared the effect of 16 methods for computing similarity matrices from the expression data; of these, simple correlation and extended Jaccard distance [12] outperformed the others and greatly exceeded the performance of single-probe association as a consequence of reduced multiple testing. As expected, performance was dependent on the number of genes in the SPs that contributed by design to the simulated phenotypes [13].

Brunel developed pathways based on probes in the GAW19 data that were annotated to genes related to BP in Gene Ontology, or to genes related to these by published evidence of protein–protein interaction. They then used independent components analysis (ICA) to derive “meta-expression” phenotypes (ICA factors). Unfortunately, the use of phenotypes derived from the real gene expression data, rather than simulated data, prevented estimation of power and type 1 error rate, so it was not possible to determine if any increase in signal from data reduction could overcome the multiple-testing burden inherent in GWAS.

### Single nucleotide variants

Valcarcel proposed 2-step association tests of a simulated BP phenotype that consisted of variant aggregation (via either SKAT or burden test) for all variants in a pathway, followed by gene-centric aggregation tests within pathways that passed a chosen significance threshold. Their pathways were synthetic (randomly chosen sets of specified numbers of genes with retention of pathways that contained at least 1 gene that was causal in the simulation). The 2-step approach was more powerful, with reasonable control of type 1 error, than a 1-step gene-centric approach because of the reduction in multiple testing by prescreening of pathways.

## Conclusions

A primary focus in the Pathway-based Analyses Group was on managing the very large dimensionality of -omics data to increase analytical power. As noted in the Introduction, pathway analysis can also be employed for biological discovery, although this use was not focal for participants in this discussion group. Methodology for this sort of discovery is also in its infancy and would also be a worthy topic for future investigation, although provision of optimal simulation data for this purpose (eg, simulated gene expression data) would be a major challenge.

Because many pathway-based approaches, including the pathway construction carried out by participants in this group, are heavily dependent on bioinformatic databases, our group discussions frequently returned to concerns about the current state of these resources, no matter how well curated – including inaccuracies, difficulty of interpretation, and (inevitably) publication bias. This suggests a possible contradiction at the heart of pathway analysis (which, regrettably, we did not discuss): many of our studies used pathway aggregation to reduce the multiple-testing burden that hampers gene and variant discovery, yet pathway construction based on prior knowledge runs the risk of constraining the scope of discovery. A possible way out of this dilemma, as noted above, is to search for empirical networks that may reveal unexpected gene interactions and thus novel biological insight.
